# Results of the Unilateral Removal of the Uterine Appendages

**Published:** 1887-07

**Authors:** 


### *RESULTS OF THE UNILATERAL REMOVAL OF THE UTERINE APPENDAGES.*

In the May number of the *American Journal of Obstetrics* there appears a lengthy communication from Lawson Tait, in which he gives the histories of twenty-six cases from whom he removed the uterine appendages upon one side only, out of the first one thousand he operated upon. Earlier in his experience he has advocated the removal of only such of the uterine appendages as he found diseased, thus leaving those that he found healthy. The results of this unilateral removal he has been able to observe, and he now has occasion to change his mind and advise the removal of both appendages, even if one is healthy. He has operated upon several a second time and removed the diseased tube, which at the first operation appeared perfectly healthy. Besides, several of the twenty-six died because they were not operated upon the second time, and the remaining eight of the twenty-six he is now satisfied need the second operation. His conclusion is more forcible to use his own language :

" So far as I know, the present contribution is the first and only evidence to be obtained on the subject, and it is quite likely that opinions may vary as to the conclusions to be derived from this, and I, for one, am quite prepared to admit that so far as it has gone by itself, it is not large enough to base any absolute conclusion upon. But the opinion which I have formed from it, and which I substantiated by more recent experience not yet mature enough for publication, and which has made an increasingly strong impression on my own mind, is, that if a patient is suffering sufficiently to justify an abdominal section for chronic inflammatory disease of the uterine appendages, and only one side found to be affected, the operation, to be of that lasting and complete benefit to the patient which we desire all operations should have, must be made bilateral. On such a point as this, of course, the desire of the patient must be paramount, as upon most others ; and if a patient placed herself under my care for such an operation, and made it an imperative condition that I should not, under any circumstances, remove the second set of appendages if they were found healthy, I should yield to her decision; but I should argue the question with her, and advise her not to subject herself to the risks of a second operation, as seems to be by far the greater tendency in unilateral operations. The list that I now present puts such incomplete operations in a very unsatisfactory light."

